# Explaining Iranian midwives’ experiences of providing healthcare services during the COVID-19 pandemic: a qualitative study

**DOI:** 10.1186/s12913-023-10265-5

**Published:** 2023-12-06

**Authors:** Sedigheh Moghasemi, Elham Adib Moghaddam, Sahar Arab

**Affiliations:** Counseling, and Reproductive Health Research Centre, Golestan University of Medical Sciences, Gorgan, Iran

**Keywords:** COVID-19, Midwifery, Pandemic, Qualitative study, Experiences, Reproductive health

## Abstract

**Background:**

COVID-19 has changed and challenged the way health and maternity care is provided. Midwives are among the first and most influential maternity care providers during the COVID-19 pandemic; however, there is inadequate information about their experiences in providing healthcare services, particularly in Iran. The present study was conducted to explain the midwives’ experiences of providing healthcare services during the COVID-19 pandemic in Gorgan.

**Methods:**

The present study was conducted qualitatively through the inductive content analysis method in 2022. Data were collected through semi-structured interviews. A total of 21 individuals were selected as participants using a purposeful method and the maximum diversity strategy.

**Results:**

Data analysis led to the emergence of 377 codes, 12 subcategories, and 3 main categories, including, the laborious occupational challenges for midwives during the pandemic, identifying and creating new opportunities for the development of the midwifery profession, and the lack of perceived organizational and social support.

**Conclusions:**

During the COVID-19 pandemic, midwives experienced various challenges in providing healthcare services, yet sacrificed themselves to perform their duties and provide quality care incessantly. The COVID-19 pandemic was a combination of laborious occupational challenges and individual and professional growth opportunities for midwives in Iran. Strong and managed organizational support is essential to overcome the crisis, maintain the workforce, and empower them to deal with future crises.

## Background

COVID-19 is an emerging and rapidly spreading disease [1], first identified in December 2019 in Wuhan, China. On February 21, 2020, Iran was recognized as one of the 31 countries affected by this virus [2]. The disease was declared a pandemic by the World Health Organization on March 11, 2020 [3]. COVID-19 has disrupted the lives of billions of people across the globe [4], fundamentally, rapidly, and unprecedentedly changed the way health and maternity care is delivered [5], and caused challenges for healthcare service delivery [6]. Despite limited information about its effect on reproductive and sexual health [7], preliminary data from the United Nations Population Fund indicates decreased healthcare services in many countries and increased challenges in providing maternity services. It also predicts an increase in maternal mortality following the COVID-19 pandemic [8, 9]. In addition, this pandemic has led to a complete ban on importing and exporting numerous basic goods and a lack of essential health items in different countries [10]. According to the International Trade Center (ITC), 23 countries have banned exports of agri-food products. For instance, on March 31, 2020, Belarus imposed restrictions on all exports of buckwheat, onions, and garlic, while at the beginning of April, Cambodia and Myanmar prohibited the export of rice [11]. Therefore, these conditions have negatively impacted health services, particularly reproductive and sexual health [10].

During pandemics, healthcare systems work at their maximum capacity and are under higher pressure [12]. Despite the COVID-19 crisis, midwifery and reproductive health services are still among the essential services to be provided [13]. Midwives, as experts in women’s reproductive and sexual health [14], have been among the first and most effective providers of midwifery care, especially during pregnancy, delivery, and postpartum during the COVID-19 pandemic [15, 16]. They also play an essential role in meeting women’s needs and individual care [17]. Midwives’ profession is emotionally challenging [18], and they experience anxiety, pain, fear, grief, excitement, and delight under normal conditions [18]. However, during the COVID-19 pandemic, midwives’ psychological challenges have become more prominent [19]. A recent study conducted in China on doctors showed a higher level of mental, emotional, and physical stress, particularly in regions with a higher prevalence of coronavirus [20]. Environmental factors and occupational stress also affect service providers’ health and the services provided by them [21].

The mental and physical fatigue experienced in the stressful conditions of the pandemic leads to midwives’ loss of motivation to provide services, disappointment, indifference, and even illness, by which service recipients will also be affected [22]. As a result, they might be afraid of providing services to poor pregnant women lacking masks due to the fear of themselves or their family members getting infected [7]. Inadequate economic situation, loss of a job or family members and friends, and disruption in the life routine and social media add to individuals’ emotional and psychological stress [23]. Since the process of providing care is influenced by workplace conditions and the culture in society [24], midwives’ experiences and perspectives can improve their care outcomes. Research supports that cultural values and norms influence healthcare experiences, specifically cultural embeddedness, cultural determinants of responsibilities or taxonomy of healthcare caregiving, and cultural values and norms underlying the decision to provide healthcare [25].

In addition, despite numerous evidence-based guidelines to guide midwives and other healthcare providers during the COVID-19 pandemic [26, 27], concerns about midwives’ practice and behavior have been raised [28], and major changes have been established in hospital-based maternity care [4], such as encouraging oxytocin use at higher doses to shorten the duration of labor, using amniotomies for dysfunctional or delayed labor, using prophylactic oxytocin during the third stage of labor to prevent hemorrhages, using early epidurals to minimize the need for general anesthesia (which risks aerosolization of the virus), limiting the second stage of labor, performing cesareans if labor had arrested after only 4 h, limiting antenatal corticosteroids after 34 weeks, judicious use of magnesium sulfate for slowing preterm labor because it can cause respiratory suppression, avoiding aggressive fluid hydration, and limiting the frequency of cervical exams [29]. During a crisis and pandemic, pregnant women’s health and fertility care are exposed to higher risk due to the lack of access to essential facilities and closer attention to those affected by the crisis [23]. Subsequently, maternal and infant mortality, which is an indicator of countries’ development, increases [30]. However, one of the main goals of sustainable development is improvements in maternal and child health care [31], which is in the scope of the midwives’ profession. Ombere likewise reports in his study that pregnant women do not refer to the hospital to receive care due to the fear of disease transmission and prefer to give birth at home, which increases the possibility of death [7].

Despite the fact that midwives are among the primary responders during the COVID-19 pandemic [1], there is limited information about their experiences in providing healthcare services during this period, particularly in Iran. Identifying the challenges, experiences, obstacles, and facilitators of providing services by midwives during the COVID-19 pandemic can serve as a practice guide in this and other crises or pandemics and help to design strategies for providing better and high-quality medical services in critical situations. Therefore, the present study was carried out to explain the experiences of midwives who provided healthcare services during the COVID-19 pandemic in Gorgan.

## Methods

### Design

Since the research team had obtained different experiences about the provision of services during the COVID-19 pandemic from midwife colleagues working in different wards, and with the assumption of achieving a better experience of providing midwifery services in unexpected future pandemics in Iran by identifying the mentioned experiences and challenges, conducted the present study using the qualitative content analysis method with an inductive approach after the approval of the Ethics Committee of Golestan University of Medical Sciences. The qualitative content analysis method includes objective and systematic concepts to explain phenomena, and in the inductive approach, the processes used to extract themes from raw data and based on valid inference and interpretation are explained [32]. Since in this study, researchers sought meanings and a relationship between concepts, this method was used.

The participants included 21 Persian-speaking midwives working in public and private healthcare centers in Golestan province in Iran, who were selected through purposeful sampling based on the strategy of maximum diversity. For the interview, a semi-structured interview guide was developed in order to make all the experiences of midwives during the COVID-19 pandemic accessible.

The research team included three members: two assistant professors in Sexual and Reproductive health (ph.D.) and one instructor of Midwifery (MSc). The interviews were conducted by the second author (Faculty member, Female, Ph.D. in Sexual and Reproductive Health), who has a long history in midwifery and reproductive health education, health service delivery, and qualitative studies.

### Setting and participants

A total of 22 interviews were conducted with 21 midwives working in the private sector (consulting center, maternity ward, and doula) and public sector (healthcare center, maternity ward, high-risk pregnancy ward, and infertility ward) across Gorgan, Iran, between January and October 2022. We chose purposive sampling to achieve representative variation with age, years of midwifery experience, highest level of education, and practice characteristics. Midwives were invited to participate by phone and were explained about the study objectives. The inclusion criteria included at least three years of work experience related to midwifery and reproductive health before the outbreak, at least one year of work during the COVID-19 pandemic and access to an online chat platform, including Google Meet or Skype, in case of conducting an online interview. It should be noted that due to the subsidence of a pandemic during the research period, all interviews were conducted in person.

We continued recruitment until saturation was reached [33]. After 20 interviews (1 interview was conducted in 2 stages due to the participant’s tiredness), we reached saturation on the level of categories and subcategories; however, we conducted two additional interviews for confirmation. Only one midwife declined to participate due to workload. To ensure full cooperation and understanding of the research aims by the participants, a consent form was administered by the second author (EAM) to each participant, and she provided information about the purpose of the study, confidentiality, and the right of the respondents not to take part in the study. Participants signed the forms after all information was provided to them. Moreover, participants’ physical and mental conditions and preparedness to participate in the interview were taken into consideration; if inappropriate, the interview was postponed to another day or was stopped and resumed another day.

### Data collection

Before the interview, participants filled out an informed consent form and a short questionnaire on their demographic characteristics. The interview was conducted by the second author (EAM) at the participant’s preferred location (midwifery practice or researcher workplace (university)). The interview started with the question, “Describe your experiences of providing midwifery services during the COVID-19 pandemic.” The interview continued based on the semi-structured interview guide. In order to deepen the interview and obtain richer data, probing questions such as “Explain more using an example?” were used based on the participants’ answers. During the interview, field notes describing the context of the interview, body language indicating the participant’s feelings, and the interview duration were recorded. Each interview lasted between 45 and 90 min.

### Data analysis

Due to the fact that qualitative research requires the researcher to immerse herself/himself in the data [34], all interviews were recorded, listened to several times, and transcribed verbatim after obtaining permission from the participants by the third author (SA). Data analysis was performed manually using codes and simultaneously with data collection using the Graneheim and Lundman method [35]. A preliminary coding scheme was developed by the first (SM) and second author (EAM) based on the framework of the interview guide and the data of three randomly chosen interviews coded by the first and second authors independently. The final coding scheme emerged during further analysis based on consensus. Transcripts were coded by the second author (EAM), who presented her analysis to the research team. Codes were grouped into subcategories and categories by examining the commonalities, differences, and relationships within and among the interviews and through reflective discussion with the research team [36].

To ensure the data trustworthiness, four criteria of credibility, dependability, confirmability, and transferability were used [37]. In order to obtain credibility, six participants were requested to check a transcription of the data and codes and confirm its accuracy. Moreover, primary codes and samples of extracting categories, subcategories, and items from the interview transcriptions for each category were provided to the external observer (Ph.D. of Reproductive Health) to ensure dependability. In order to establish confirmability, the transcription of eleven interviews and extracted codes and categories were provided to the researcher’s colleagues and three reproductive Health and Midwifery specialists who were not involved in the study. They were requested to check the accuracy of the data coding process. To ensure transferability, we used verbatim transcripts and thick descriptions in data analysis. The writing of this article was guided by the consolidated criteria for reporting qualitative research (COREQ) [38].

## Results

Of the 22 midwives invited, 21 participated in the study. Participants’ background characteristics are presented in Table 1. The analysis of interviews with participants with work history ranging from 5 to 29 years was associated with the extraction of 377 inferential codes. After merging repeated codes with the same concept, 12 subcategories, and 3 main categories were achieved (Table 2). Extracting categories from meaning units are presented in Table 3.

**Table 1:** Participants’ characteristics

Participant number	Work experience (year)	Education	Workplace
1	15	Bachelor of Midwifery /Master of Psychology	Private sector (consulting center)
2	9	Bachelor of Midwifery	Private sector (maternity ward)
3	29	Bachelor of Midwifery	Private sector (maternity ward manager)
4	14	Master of midwifery	Public sector (healthcare center)
5	11	Bachelor of Midwifery	Public sector (healthcare center)
6	14	Bachelor of Midwifery	Public sector (healthcare center)
7	15	Bachelor of Midwifery	Public sector (healthcare center)
8	5	Bachelor of Midwifery	Private sector (doula)
9	28	Bachelor of Midwifery	Public sector (maternity ward manager)
10	20	Bachelor of Midwifery	Public sector (maternity staff)/private sector
11	24	Bachelor of Midwifery	Public sector (high-risk pregnancy ward manager)
12	25	Master of midwifery	Public sector (infertility ward manager)
13	27	Bachelor of Midwifery /Master of Psychology	private sector (office)/ public sector (maternity staff)
14	29	Bachelor of Midwifery	Public sector (high-risk pregnancy ward manager)
15	8	Master in counseling in midwifery	Public sector (healthcare center)
16	25	Bachelor of Midwifery	Private sector (maternity ward)
17	9	Bachelor of Midwifery	Public sector (healthcare center)
18	9	Bachelor of Midwifery	Private sector (maternity ward)
19	15	Bachelor of Midwifery	Private sector (doula)
20	20	Bachelor of Midwifery	Public sector (maternity ward manager)
21	12	Bachelor of Midwifery	Private sector (doula)

**Table 2:** Categories and subcategories

Subcategory	Category
Deprivation of social and family support Workplace unsafety Doulas’ Job restrictions Low rate of risk perceived by clients Increased workload without perks Care-therapeutic confusion in midwifery Threats to mental health	Laborious occupational challenges for midwives during the pandemic
Creating a supportive atmosphere in the workplace New job opportunities for midwives in social media Self-motivation to provide quality midwifery care	Identifying and creating new opportunities for the development of midwifery profession
Lack of perceived social-organizational gratitude Lack of perceived social support	Lack of perceived organizational and social support

**Table 3:** Extracting categories from meaning units

Category	Subcategory	An example of primary codes	An example of Quotes
Identifying and creating new opportunities for the development of midwifery profession	Creating a supportive atmosphere in the workplace	Strengthening cooperation, empathy, and altruism among colleagues	When one of the colleagues got infected, another tried to cover her place until that person fully recovered. If one of us were tired, we would do each other’s tasks without any expectations, even though we were all tired (Participant No.5).
		consolidating teamwork	The COVID-19 pandemic was a good experience overall and made us stronger. It strengthened our teamwork in critical situations. The experts also accepted that it was really necessary to work as a team, and in such a situation, they couldn’t succeed without midwives.
	Self-motivation to provide quality midwifery care	Increasing accuracy in performing procedures (vital signs, etc.) and practice	During the screening, we usually discovered other things. We interpreted the tests more carefully. Once we identified a patient with 2.9 creatinine. The patient wasn’t infected with COVID-19, but I might not have read his test so carefully if it wasn’t during the pandemic (Participant No.18).

### Laborious occupational challenges for midwives during the pandemic

The midwives’ statements indicated the laborious challenges of providing healthcare services during the COVID-19 pandemic. This category resulted from subcategories of deprivation of social and family support, workplace unsafety, job restrictions of doulas, low rate of risk perceived by clients, increased workload without perks, care-treatment confusion in midwifery, and threats to mental health.

Deprivation of social and family support was one of the concerns caused by the pandemic. Participant No. 18 stated: *“Many people were afraid of us working in the hospital. When I needed a call taxi service, if they knew I worked in the hospital, they declined my call. So I talked to the company manager once, and he said that the company wouldn’t provide services to hospital personnel. I justified it and asked him how he would know the other person they gave service wasn’t infected. Would they take a test to see if they weren’t a carrier or not infected? I got so upset, and I never asked them for a car again.”*

The pandemic led to workplace safety in some centers and unsafety in others regarding coronavirus infection. Private centers were considered safer due to not admitting infected patients. On the other hand, selected public and related centers were considered unsafe due to the referral of infected individuals. In this regard, Participant No. 9 stated: *“Our hospital was a center for COVID-19 patients. Our personnel were completely exposed to infection, especially at the beginning when we had a shortage of personal protective equipment. But the condition was much better in private centers. Because patients suspected of being infected in screening were also referred to us, they were normally less susceptible than us.”*

The pandemic imposed a restriction on the work conditions of doulas, who were not allowed to enter hospitals during peak times. In addition, due to the nature of the coronavirus disease and its respiratory transmission, some interventions used by doulas, such as respiratory techniques, were highly limited. Participant No. 8 said: *“You couldn’t educate things such as inhaling and exhaling. The mother was unwilling because of the coronavirus, and I, the midwife, was terrified. Well, there was fear of losing life; fear of death.”*

Another challenge midwives experienced in providing services to clients was the low level of risk perceived by clients regarding infection. Participant No. 7 stated: *“Making clients wear a mask and also coming inside without a companion was really a challenge; we kept explaining that it was first for their benefit, then for us. But people were strongly resistant. They said they wouldn’t get it or believed there was no virus; maybe it was because they couldn’t afford to buy masks, which were very expensive.”*

The pandemic outbreak led to an increase in the workload of all health service providers, including midwives. Participant No. 17 stated: *“Our center was selected for COVID-19. Different patients were referred there. We are midwives, but we also did the nurses’ tasks; for example, we did an IV insertion for a patient with typical symptoms of coronavirus disease and had to be admitted to the hospital. In addition, some days, we had to go to the vaccine centers as vaccinators, and we couldn’t deal with our own tasks, or in my absence, the workload at the center was taken by my colleague, who was alone that day.”*

The unknown nature of the disease at the beginning of the outbreak, the announcement of multiple instructions, the lack of a uniform protocol, the lack of consensus between physicians regarding the final decision for the patient, diagnostic and therapeutic limitations during pregnancy and the fear of doctors and clients being infected, were among midwives’ care challenges. The unknown nature of the disease had caused a large number of patients to refer to private hospitals. For example, participant No. 3 stated: *“The number of mothers referring to the private sector was very high because they believed that the public centers were contaminated. Those who believed they had these symptoms denied it and visited the private sector. So we fell between two stools; there was pressure on us”.* Or regarding the lack of agreement between doctors about making the final decision for the patient, the same participant stated: *“The doctors were talking equivocally; the gynecologists wanted their patients to be hospitalized and preferred them to have a cesarean section, but the infectious disease doctors believed it wasn’t expedient to hospitalize them there.”*

The anxiety of infection and the fear of death among the medical staff, including midwives, was extremely high, especially at the beginning of the pandemic. In this regard, participant No. 18 stated: *“Every day, when I went home, I felt like I was infected. I was always anxious. Because the patients and we believed that anyone who gets infected during the peak will die.”*

Regarding the high mental pressure, participant No. 2 said: *“Six out of ten of us got infected with coronavirus. Only 4 staff were left uninfected in the maternity ward. Well, there were at least 30 patients in each shift, 30 patients with one or two staff. All patients had a lot of tasks to be done for them. Well, it was a lot of mental pressure for us; we were anxious. The patients were also anxious, so we had to try not to transfer our anxiety to them; we also calmed them down.”*

Some midwives stated they were obsessed with washing due to the fear of infection. In this regard, participant No. 4 stated: *“I used to wash my and my husband’s clothes in the washing machine every day. Before COVID-19, when we reached home, we used to wash our hands and face, but after the coronavirus disease, every day we come home, we must take a shower. I’ve got obsessed.”*

### Identifying and creating new opportunities for the development of the midwifery profession

The pandemic outbreak led to the flourishing of professional opportunities, precision in practice, and higher empathy among midwifery staff. This category was derived from the subcategories: ‘creating a supportive atmosphere in the workplace,’ ‘new career opportunities for midwives in social media,’ and ‘self-motivation to provide quality midwifery care.’

Consolidation of teamwork, cooperation, empathy, and altruism among colleagues were the advantages of the pandemic. Participant No. 13 stated: *“When one of the colleagues got infected, another tried to cover her place until that person fully recovered. If one of us were tired, we would do her tasks without any expectations, even though we were all tired.”*

The pandemic outbreak, quarantine, and subsequent traffic restrictions helped midwives to optimally use their free time. Some midwives had an opportunity to share their training online at no charge. Some used this opportunity and offered consultations and visits online. In this regard, participant No. 6 said: *“I started childbirth preparation classes on WhatsApp. Well, it was a very nice experience; it was very well-received; members added their friends from all over the country to my group, and I answered all their questions related to midwifery.”* Participant No. 1 stated: *“I used the opportunity to hold joint Instagram live sessions with doctors to educate people and answer their questions. For example, I used to read the content printed by a doctor because I believed that maybe everyone doesn’t have access to it. I went live on Instagram; questions and answers about COVID-19, for example, pregnancy, coronavirus disease, and Gynecological problems.”*

The COVID-19 pandemic led to higher motivation in midwives to study and provide better quality services. They identified other disorders and diseases due to the need to screen patients for hospitalization in COVID-19 and non-COVID-19 wards. In addition, they observed the principle of appointment with the shortest waiting time for clients to avoid congestion in the service-providing centers. In this regard, Participant No. 2 said: *“During the screening, we usually discovered other things. We interpreted the tests more carefully. Once we identified a patient with 2.9 creatinine. The patient wasn’t infected with COVID-19, but I might not have read hes test so carefully if it wasn’t during the pandemic.”*

#### Lack of perceived organizational and social support

Lack of perceived social-organizational gratitude and lack of perceived social support were the two subcategories of this category. The need to receive appreciation and material support and the value their lives were the issues that the midwives mentioned frequently. Despite the increase in midwives’ workload and their industrious efforts, material and spiritual incentives were not considered for them by the healthcare system. In this regard, Participant No. 10 stated: *“We worked tirelessly. I couldn’t see my son for a whole month because of my job and family circumstances. But it seems that our efforts and the pressure we endured weren’t noticed at all; officials didn’t even simply thank us or didn’t consider any measures for us, such as special leave or compensation for expenses.”*

Taking into account special measures for active midwives during the pandemic and understanding the pandemic conditions by high-ranking officials were among the midwives’ professional needs. In this regard, participant No. 15 stated: *“Our officials never perceived the condition. Whenever they came, instead of appreciating our effort, they just told us that we didn’t care well or why there were so many non-cooperation letters among pregnant women. Well, no matter how much we explained about disinfecting everywhere, pregnant women were afraid to come to the health center to control the baby’s heart rate. There were no other mothers when officials came, but they wouldn’t still be justified. Managers and supervisors never complimented us. They only condemned us for poor care or incomplete tasks. Our competencies and efforts were never noticed.”* Expecting clients to understand critical conditions and their cooperation with the medical staff and compliance with health protocols was considered as social support for midwives and other healthcare workers. In this regard, Participant No.14 stated: *“Some people came to the hospital without wearing masks. I used to say, “without exception, the mother who doesn’t have a mask should go out. We are working honestly, you must be honest with us, otherwise we will not accept you.”*

## Discussion

This study aimed to explain the experiences of Iranian midwives in providing healthcare services during the COVID-19 pandemic. The findings showed that midwives’ experiences were noteworthy in three dimensions: laborious occupational challenges for midwives, identifying and creating new opportunities for the development of the midwifery profession, and lack of perceived organizational and social support.

The rapidly evolving pandemic situation, the need to change, and contradictions in patient management [39] are associated with midwives’ confusion and increased workload [40]. The necessity of congregating patients in selected hospitals in order to prevent the spread of the disease, the possibility of concealment of patients regarding the infection, the lack of personal protective equipment for the general public [41], and the low perceived risk of infection and death among clients, play an important role in the insecurity of the COVID-19 centers [40]. In addition, the unknown nature of the disease leads to midwives’ fear and anxiety [20], decreased efficiency, and double fatigue [1]. Medical staff’s anxiety and concerns decrease by increasing the level of their knowledge of the ways to deal with the pandemic [1].

The contagious nature of the coronavirus and the need to observe social distancing and prevent the presence of non-essential individuals in the patient’s bedside and wards led to restrictions on the employment of some midwives, including doulas. This finding is consistent with other studies. In Rivera’s study in the United States, the change in hospital care during the COVID-19 pandemic had a direct impact on the care of doulas [4]. In Stulz’s study, social distancing prevented quality midwifery care [40]. In fact, limiting the presence of doulas leads to mothers’ deprivation of the support services provided by them, which increases their fear and worries [42], increases the possibility of mood disorders after childbirth, decreases mental health [43], and increases elective cesarean delivery [44]. Since most delivery rooms are LDR (Labor, Delivery, Recovery), adopting strict policies can deprive mothers of some of their human rights and is contrary to scientific and ethical principles [45].

The community’s fear of contracting COVID-19 after contacting the medical staff led to the deprivation of midwives from some social services. Moreover, due to the nature of their job, midwives had minimized and sometimes eliminated their family contacts [40]; all of these isolating factors were a threat to the mental health of midwives and even their families. Fear, anxiety, and worry in the medical staff, including midwives, have been reported in other studies as well [40, 43]. The fear of COVID-19 was stronger at the beginning of the crisis and resulted from the unknown nature of the disease, rapid changes, lack of personal protective equipment, and limited human resources [40]. The most common cause of fear was the transmission of the disease to family members [46]. Considering the necessity of maintaining midwives’ mental health during crises, including the COVID-19 pandemic [46], psychological interventions, including providing an opportunity for midwives to express their experiences and needs [47] and the chance of benefiting from face-to-face or online private consultations should be considered [48].

The pandemic has led to a supportive atmosphere in the workplace, creating new career opportunities in cyberspace and a high motivation for midwives to provide quality midwifery care. Strengthening the teamwork and coordination of the medical staff was reported in Hantooshzadeh’s study, which examined the experiences of health service providers regarding maternal and newborn care in Iran [39]. In line with the present study, Fumagalli reported the development of positive occupational aspects related to the COVID-19 pandemic, including individual and professional growth, improving communication skills, strengthening interpersonal trust, a sense of competence, and strengthening teamwork and a supportive atmosphere in midwives in Italy [49].

Since midwives were one of the most important providers of services to society, particularly mothers and infants, they made an effort to keep their information up-to-date by increasing reading and searching on the Internet so that they could answer clients’ questions. In Stulz’s study, studying more to answer women’s questions about the coronavirus showed the importance of this issue for midwives [40]. The development of such competencies during the pandemic has led to the individual and professional growth of midwives, and as a result, it can be effective in providing more quality services and dignified midwifery care [50].

Lack of organizational-social gratitude and perceived social support constituted the ‘organizational and social support perceived by midwives’ dimension. It is inevitable to take some measures in the healthcare systems to maintain and improve the health and productivity of human resources who are working in the front line of the fight against coronavirus worldwide [42]. Considering the job difficulty and the critical conditions, midwives worked together with other members of the medical staff and were on the front line of the fight. In addition to making a great deal of effort in maintaining and promoting the health of mothers and infants, they played a role in providing services for patients with COVID-19 in medical centers. However, it seemed that their efforts were not recognized or valued by the relevant organizations. They also needed recognition and expected the healthcare system to value their lives. They also expected that due to the high workload, material and spiritual benefits would be considered for them [39, 42] since most of them or their loved ones became infected or were deprived of visiting their families and children due to working at the front line [40]. As a result, it is indispensable for government organizations, midwifery associations, and other stakeholders to provide adequate support to midwives for their welfare [51]. In fact, midwives, similar to other medical staff, expect to be “heard,” “protected,” “prepared,” “supported,” and “taken into account” [52].

### Strengths and limitations of the study

Few studies have investigated the experiences of midwives working in different healthcare sectors, both private and public, during the COVID-19 pandemic in Iran; therefore, the findings of the present study have revealed important dimensions of the challenges of midwifery care and midwives in Iran. In the present study, interviews with midwives were conducted when the peak of the COVID-19 pandemic and the disease had almost subsided, which may have led to the dimming of midwives’ memories of their experiences. However, an attempt was made to overcome this problem by conducting numerous interviews with key individuals and midwives working in different wards. Moreover, an effort was made to extract significant points by accompanying the interviewees and considering all the details and memories related to the research topic as important.

## Conclusion

The COVID-19 pandemic was a combination of laborious occupational challenges and personal and professional growth opportunities for midwives in Iran. Strong and managed organizational support is necessary to overcome the crisis, maintain and sustain the midwifery staffing, and empower them to deal with future crises. Improving the provision of personal protective equipment, adopting strategies to improve mental health, properly managing staff in crisis, and considering special measures and various incentives in crises for medical staff, including midwives, are of particular importance. In addition, educating the general public in order to take protective measures and promote a culture of appreciation for midwives and medical staff can reduce the work and psychological burden caused by the crisis.

## Data Availability

The datasets generated and/or analyzed during the current study are not publicly available to protect study participant privacy but are available from the corresponding author upon reasonable request.

## Abbreviations

ITC: The International Trade Center
LDR: Labor, Delivery, Recovery
